# ABSSeq: a new RNA-Seq analysis method based on modelling absolute expression differences

**DOI:** 10.1186/s12864-016-2848-2

**Published:** 2016-08-04

**Authors:** Wentao Yang, Philip C. Rosenstiel, Hinrich Schulenburg

**Affiliations:** 1 Evolutionary Ecology and Genetics, Zoological Institute, CAU Kiel, Am Botanischen Garten 9, 24118 Kiel, Germany; 2 Centre for Molecular Biology, Institute for Clinical Molecular Biology, CAU Kiel, Am Botanischen Garten 11, 24118 Kiel, Germany

**Keywords:** RNA-Seq, Transcriptome analysis, Differential gene expression, ABSSeq, Negative binomial distribution

## Abstract

**Background:**

The recent advances in next generation sequencing technology have made the sequencing of RNA (i.e., RNA-Seq) an extemely popular approach for gene expression analysis. Identification of significant differential expression represents a crucial initial step in these analyses, on which most subsequent inferences of biological functions are built. Yet, for identification of these subsequently analysed genes, most studies use an additional minimal threshold of differential expression that is not captured by the applied statistical procedures.

**Results:**

Here we introduce a new analysis approach, ABSSeq, which uses a negative binomal distribution to model absolute expression differences between conditions, taking into account variations across genes and samples as well as magnitude of differences. In comparison to alternative methods, ABSSeq shows higher performance on controling type I error rate and at least a similar ability to correctly identify differentially expressed genes.

**Conclusions:**

ABSSeq specifically considers the overall magnitude of expression differences, which enhances the power in detecting truly differentially expressed genes by reducing false positives at both very low and high expression level. In addition, ABSSeq offers to calculate shrinkage of fold change to facilitate gene ranking and effective outlier detection.

**Electronic supplementary material:**

The online version of this article (doi:10.1186/s12864-016-2848-2) contains supplementary material, which is available to authorized users.

## Background

Transcriptome studies usually aim at understanding inducible biological functions through an analysis of differential gene expression (DE). Since relatively recently, the variation in gene expression is commonly studied through RNA sequencing or RNA-Seq, based on next generation sequencing (NGS) technologies. In these study approaches, DE is usually inferred from comparison of two different treatments, developmental stages, or different tissues. A key step in these analyses is the reliable identification of significant DE. Most current statistical approaches employ a probabilistic model, such as the Negative Binomial (NB) [1–3], Poisson [4], the Generalized Poisson (GP) model [5], and use information on gene expression variation in the data to account for ambiguity caused by sample size, biological and technical biases, overall levels of expression and the presence of outliers. DE inference is usually based on the null hypothesis that the means of read counts among conditions are the same or follow the same distribution. These tests neglect the magnitude of encountered differences and might report statistically highly significant DE with arbitrarily small fold change, at least if the number of sequencing counts is large enough [6, 7]. However, small fold changes may represent artifacts and often cannot be validated experimentally (e.g., through Realtime PCR approaches or functional genetic analysis). Thus, they might not be worth further investigation. A currently common solution is sought by combining the statistical indication (i.e., an FDR-adjusted *p*-value) with a specified minimum fold change [8, 9]. This approach has the possible problem of a high number of identified candidate genes with low count numbers (which may produce high fold change by chance) and its dependence on an arbitrarily chosen fold-change cut-off value.

An alternative approach has so far only been established for ChIP-Seq data and relies on an analysis of count differences between test and reference conditions [10, 11]. In this case, the statistics are based on a measure that considers the magnitude of count differences and the level of expression variation across replicates with the effect that genes with only minor expression levels and only small fold change are selected against. In consideration of such potential advantages, such an approach may prove useful for reliable DE identification in RNA-Seq data.

Here, we introduce ABSSeq (i.e., differential expression analysis of ABSolute differences of RNA-Seq data), which employs an NB distribution to model count differences between conditions. It permits testing the magnitude of observed count differences taking into consideration background expression level variation. In particular, ABSSeq accounts for heterogeneous dispersions in expression level across genes by adding expected values (pseudocounts) to reads count according to the smoothed mean-variance relationship [1], which thus adjusts parameters in the NB distribution (mean and size). In addition, ABSSeq imposes a penalty on the dispersion estimation, it uses a new outlier detection strategy, and it also inroduces a procedure for shrinkage of fold change to disfavor identification of candidate genes with abnormal high dispersions and extremely low expression. Using real and simulated datasets, we demonstrate that our method is highly efficient in reducing the false discovery rate (FDR) and thus in identifying truly differentially expressed genes in RNA-Seq data. It therefore shows an at least similar performance than several frequently used, alternative approaches like those implemented in the software packages DESeq [1], DESeq2 [1, 12], edgeR [3, 13] (referred as edgeR-robust when applied on data set with outliers), limma [14, 15] (referred to as Voom), baySeq [2], and EBSeq [16].

## Implementation

ABSSeq has been implemented in the software package ABSSeq for the cross-platform environment R [17]. ABSSeq is released under the GPL-3 license as part of the Bioconductor project [18] at URL: http://bioconductor.org/packages/devel/bioc/html/ABSSeq.html.

## Results and discussion

We firstly introduce our approach with the help of the modencodefly and ABRF datasets (see Datasets). Thereafter, performance of our method is compared with that of several previously developed and currently popular methods (always used under default settings, for example limma under eBayes settings and the TMM normalization for limma and edgeR; see Additional file 1), including one, EBSeq, which allows to evaluate DE at both transcript and also gene level [16]. We exlcude Cuffdiff2 [19] from our assessment because it was previously compared with the other available approaches and generally found to produce higher rates of false positives without an increase in sensitivity [20]. Method evaluation is based on two types of data sets. On the one hand, we use simulated data, for which data structure can be efficiently controlled and which have been widely used to evalute methods of differential expression analysis [2, 7, 21–24]. We use the same strategy and identical simulated data sets as Soneson et al. [7] and compare method performance according to two criteria: (i) the ability to control type I error rates; and (ii) the ability to rank truly DE genes ahead of non-DE ones. On the other hand, we also evaluate our approach with the help of real data sets, as described in more detail below.

### Control of type I error rate

**Fig. 1:**
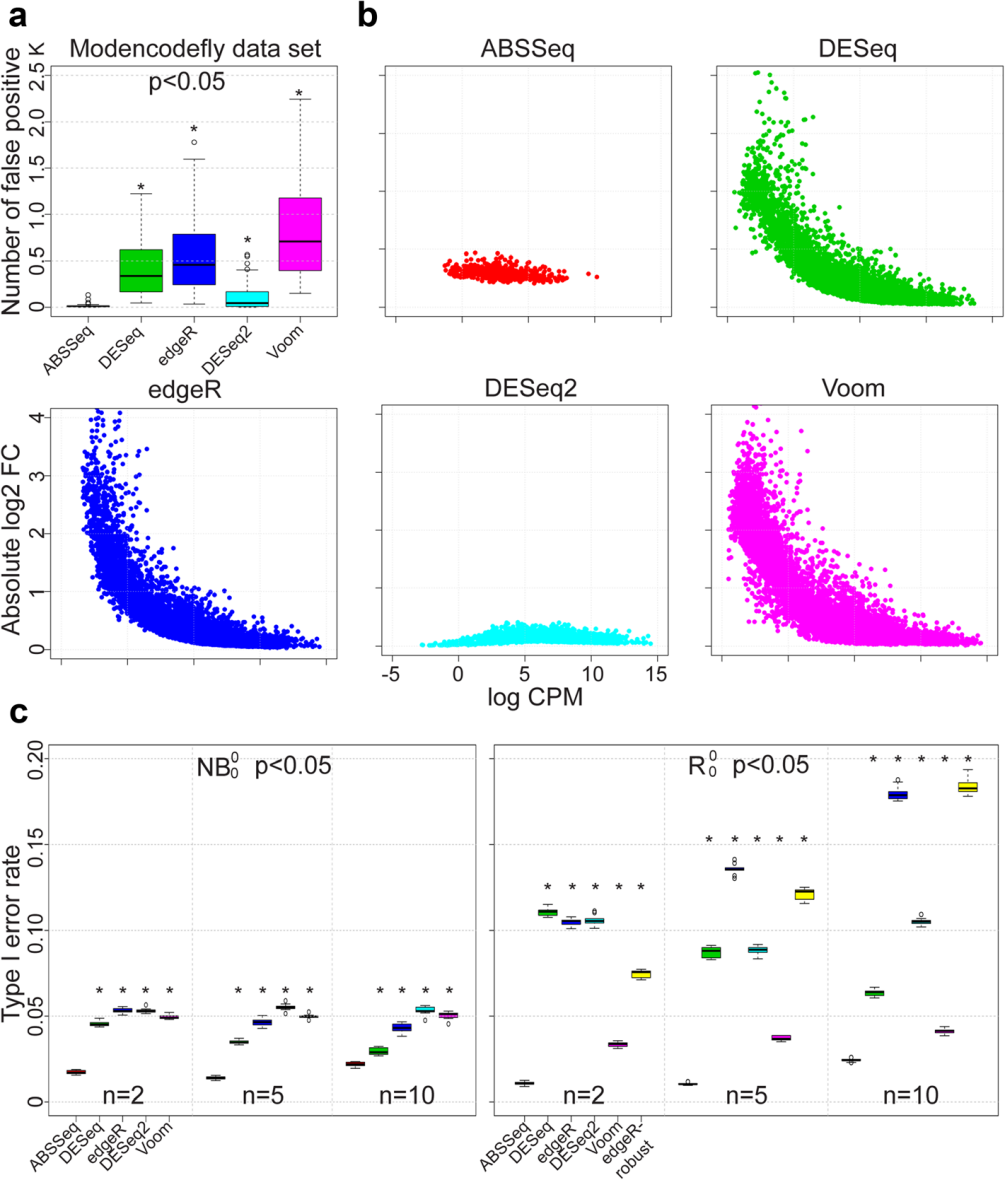
Method-dependent variation in type I error. Type I error rates for ABSSeq and five alternative methods using the modencodefly real data set (**a**) and two simulation settings: Negative Binomial (NB, c left panel), and NB with random outliers (R, c right panel). **b** Points show the absolute log fold change (FC, y-axis) distribution of false positives against the expression level (logCPM, x-axis). **c** Each boxplot summarizes the type I error rates across 10 independent simulated data sets. Asterisk indicates a statistically significant difference in type I error between ABSSeq and any of the other methods. n indicates the number of RNA replicates considered in each case (ranging from 2 to 10). Under all conditions, ABSSeq reduced the type I error rate

Minimizing the type I error rate (i.e., the null hypothesis is falsely rejected) or false positive rate is a primary goal of differential expression analysis [20, 25]. Type I error is often introduced by under-estimation of disperson in RNA-Seq data and occurs at genes with very low or high counts [20]. We thus compare the ability of the alternative approaches to control type I error rates, using two real data sets and also the simulated data sets from Soneson et al. [7]. DE genes are defined by a *p*-value cutoff of 0.05 for each method except baySeq and EBSeq, which are excluded from this comparison since they report DE by posterior probalilities instead of a *p*-value. The simulated datasets are assumed to lack DE genes, facilitating computation of the type I error rate by dividing the number of DE genes identified by each method with the total number of genes. Figure 1 summarizes the results from the modencodefly data set (Fig. 1a and 1b) and two different simulation settings (Fig. 1c), including data sets of various replicate sample sizes and, in each case, ten independent repetitions (see also Additional file 2). Additional file 3 shows the results for the ABRF data set.

The first comparison is based on a real data set for the fruitfly *Drosophila melanogaster*, the modencodefly data set [26], which characterizes the developmental transcriptome across 30 distinct stages (conditions) with technical replicates ranging from 4 to 6. We randomly select 4 replicates for each condition and separate them into two groups, which should thus only be characterized by stochastic variations but not true DE. The results of our analysis is summarized in Fig. 1a. At the *p*-value cutoff of 0.05, ABSSeq identifies an average of 17 DE genes and thus significantly fewer DE genes than all alternative methods (Wilcoxon rank test, *p* < 0.01). DESeq2 also performs well on this real data set, while the highest type I error rate is obtained for limma (873 identified cases of DE).

Next, we examined the distribution of false positives along absolute log2 fold change and expression level (log of average counts per million, logCPM, calculated by edgeR) using the data from Fig. 1a. As shown in Fig. 1b, false positives with low logCPM (x-axis) tend to have a high fold change (DESeq, edgeR and Voom), and vice versa. This skewed distribution is very similar to the quadratic mean-variance relationship [1, 5, 15], suggesting there might be a general under-estimation of variance or dispersion for these methods. In contrast, ABSSeq and DESeq2 both shrink the fold change according to variance. As a consequnce, they both exhibit a pronnounced reduction of false positives at low expression (logCPM < 0). Morever, ABSSeq also reduces false positives at high expression level (logCPM > 10), which likely have a very low smoothed dispersion [1] and are often inferred to be highly statistically significant but show only very small fold change.

As the modencodefly data set only allows us to consider two replicates per group and condition, the resulting statistical power may be limited. Therefore, we repeated this assessment using another real data set, the ABRF data set [27] (see Datasets in Methods section), which is based on an RNA-Seq analysis of the same two samples across three independent laboraties and thus comprises for each sample three replicates that should only show variation caused by differences among the laboratories such as library preparation methods or sample processing procedures (6 comparisons in total, Additional file 3). The analysis of the ABFR data set confirms the previous results. It demonstrates that ABSSeq produces the smallest number of false positives and especially reduces fold change for the genes with generally low expression.

Overall, the results from the two real data sets suggest that ABSSeq has the ability to handle very small expression changes by considering the magnitude of absolute differences and penalizing the estimated dispersion (See Methods). Our results also suggest that the alternative methods should allow enhanced reduction of the type I error rate if combined with additional filtering approaches, such as usage of a fold-change cut-off as discussed in [28, 29] and also further below.

In addition to the two real data sets, we also compare the ability of the alternative approaches to control type I error rates on simulated data (Fig. 1c). Generally, all methods are able to control type I error rate under 0.05 when applied on the NB distributed data (Fig. 1c left panel, denoted NB_0_^0^, 0 indicates the number of up or down-regulated genes) but exhibit high diversity on the NB distributed data with randomly introduced outliers (abnormally high counts, multiplying a randomly generated factor between 5 and 10 with counts of genes randomly selected with a probability of 0.05, denoted by S_0_^0^, Fig. 1c right panel). As already highlighted in [7], DESeq has excellent power to control type I error rates on NB_0_^0^. The performance of Voom is relatively unaffected by sample size and outliers, implying advantages of log-transformation on dealing with high value outliers. In contrast, edgeR does not control type I error rates efficiently when applied on data with outliers. Since both DESeq2 and edgeR-robust integrate strategies to handle outliers, they expectedly reduce the type I error rate on S0 0, especially when compared to the earlier program versions (e.g., DESeq at *n* = 10 or edgeR at *n* = 2 or 5). ABSSeq performs best in both cases (Tukey's, *p* < 1.0e-3), but slightly decreases its performance with increasing sample size (*n* = 10).

Taken together, ABSSeq is able to efficiently control type I error rates for the real and simulated data sets (Fig. 1a-c) and it also reduces type I error rate at both low and high expression levels. In addition, outliers impact the ability of controlling type I error rate for most methods except ABSSeq and Voom, which might be caused by shrinkage of the observed dispersion (edgeR, edgeR-robust and DESeq2) or replacing the observed with a smoothed dispersion (DESeq). In contrast, ABSSeq uses the observed dispersion directly, apparently enhancing control of type I errors to a rate of below 0.05.

### Discrimination of DE versus non-DE genes in simulation studies

**Fig. 2:**
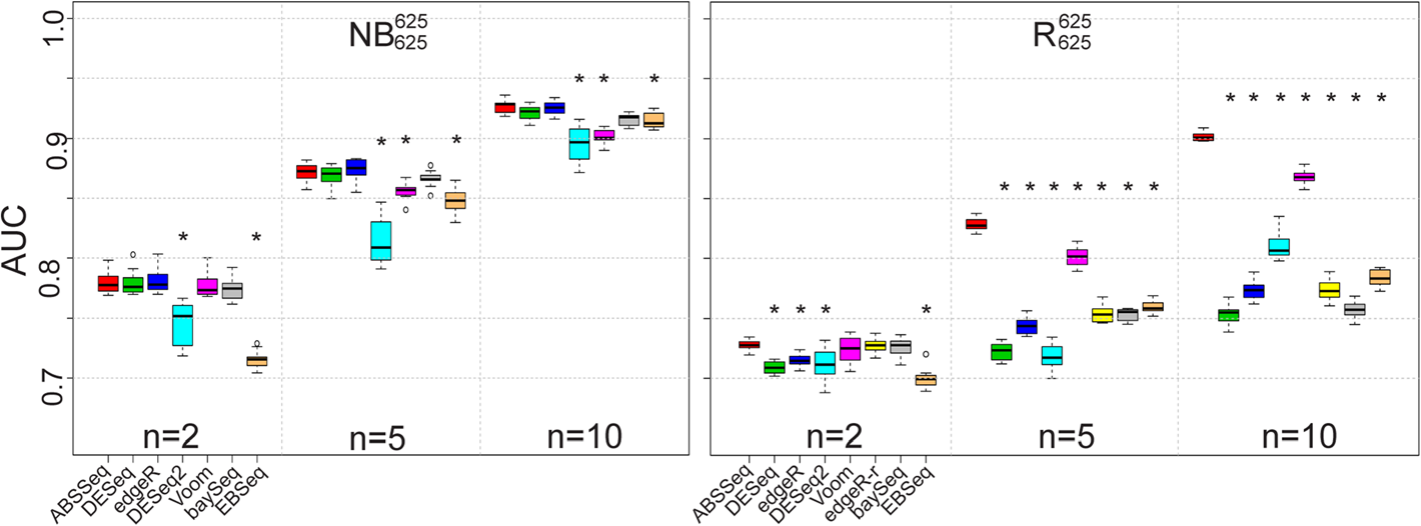
AUC comparison on simulated data. Area under the curve (AUC) for ABSSeq and five alternative methods under two simulation settings: Negative Binomial (NB, *left panel*) and NB with random outliers (R, *right panel*). Each boxplot summarizes the AUCs across 10 independently simulated data sets. Asterisk indicates a statistically significant difference in AUC between ABSSeq and any of the other methods. n indicates the number of considered RNA-Seq replicates, from 2 to 10. Under all conditions, ABSSeq is highly effective in correctly identifying differentially expressed genes

An ideal DE inference method should be more sensitive to DE than non-DE genes, that is, it should be able to discriminate true DE genes against non-DE ones. Here, we evaluate the discriminative power of ABSSeq and other selected methods in terms of the true and false positive rates and also the area under Receiver Operating Characteristic (ROC) curve (AUC), using again the simulated data and general approach of Soneson et al. [7]. The AUC was shown repeatedly to be informative as a measure of the overall discriminative performance of a method [30–32]. In particular, for our comparison, we extract a set of genes from the simulated data set using a given *p*-value or posterior probability (baySeq) threshold. Thereafter, the obtained genes are divided into a truly positive group and a truly negative group according to pre-defined DE genes in the simulated data. This information then allows us to calculate the true positive and the false positive rate for all possible thresholds, construct ROC curves and compute AUCs using the ROC package in Bioconductor [18]. For all simulations, we choose 10 % of the 12,500 genes as DE and symmetrically divide them into up- and down-regulated genes (e.g., 625 up- and 625 down-regulated genes, indicated below by super- and subscripts, respectively). We summarize the results using boxplots for four different simulation settings, including data sets with various replicate sample sizes and, in each case, ten independent repetitions (Fig. 2, Additional file 2).

When applied on the data set simulated using the NB distribution (denoted by NB_625_^625^, where the super- and subscripts indicate the number of up- and down-regulated genes, respectively; Fig. 2 left), ABSSeq always performs at least as good as the alternative methods at the considered replicate sample sizes (denoted by n). EBSeq performs worse than the other approaches when applied on data with small sample size (*n* = 2). The performance of ABSSeq and the other methods are generally improved as the sample size increases, revealing a positive power of sample size on identifying true DEs. Overall, these results suggest that our NB model fits the over-dispersion data at least as well as the NB model implemented in other methods.

We next test the influence of outliers, which we introduce into the NB distributed data using a similar approach as above (denoted by R_625_^625^, Fig. 2 right) and which may show abnormally high counts, resulting in high fold changes and also false postives. For these simulated data sets, ABSSeq shows an advantage (Tukey's, *p* < 0.01) at all replicate sizes, especially for the R_625_^625^ data set (Tukey's, *p* < 1.0e-6) whose AUC is even greater than 0.9 at *n* = 10 (Fig. 2c, 2d). This result indicates that ABSSeq outlier detection is efficient. Interestingly, performance of the alternative methods also shows substantial variability. For example, Voom generally performs better at large sample size (i.e. higher AUC in R_625_^625^ except ABSSeq), but similar at small sample size with other methods; DESeq2 performs better at *n* = 10 due to outlier detection but worse at *n* = 2 and *n* = 5 (*n* ≥ 7 required for outlier detection); baySeq shows little improvement in performance as the sample size increases for the R_625_^625^ data set; EBSeq shows lowest AUC at *n* = 2 and improves performance at large sample sizes (*n* = 5 or 10); edgeR-robust (denoted by edgeR-r) shows an improved ability to handle outliers at small sample size (*n* = 2 or 5) compared to edgeR.

Overall, ABSSeq is at least as good as alternative methods in discriminating between DE and non-DE genes, it is highly robust towards outliers at all sample sizes, while increasing the sample size improves the discriminative performance for all methods. The high performance of ABSSeq on the outlier data sets supports the efficiency of the implemented approach based on moderated median absolute deviation (MAD) in outlier detection even at small sample size (see Methods). Together with the results in Fig. 1, our model on count differences seems to perform at least as good as other models using NB distributed data.

### Differential expression analysis on qRT-PCR validated real data

As simulated data are by nature artificial, we further evaluate method performance on real data sets. The first of these relate to the MAQC study, for which RNA-Seq-identified DE genes were validated by quantitative reverse transcription PCR (qRT-PCR) [33] based on the commercially available TaqMan and PrimePCR methodologies. Although there is no single “gold standard” for assessment of RNA-Seq data reliability [29], qRT-PCR based methods have widely been proposed and applied as a validation tool for DE results from both microarray [34] and RNA-Seq studies [35]. Here, we analysed two qRT-PCR validated data sets from the MAQC study: the TaqMan data set from MAQC-I, which included an assessment of a very small fraction of the total genes (1044 out of more than 50,000 genes from hg19 annotation) and may thus be subject to biases, and additionally the PrimePCR data set from SEQC (equivalent to MAQC-III), which covers more than 20,000 genes [29]. These two data sets were used to derive ROC curves and AUC measures for the compared analysis methods. We consider this approach to provide at least an indication of the reliability and sensitivity of the analysis approach. We here follow the general strategy from [36] and [20] and divide the TaqMan and PrimePCR gene sets into a DE (true positive) group and a non-DE (false positive) group based on whether their absolute log fold change (logFC) is larger or smaller than a defined threshold. We use a logFC threshold of 0.5 (1.4 fold change) to derive ROC curves.

**Fig. 3:**
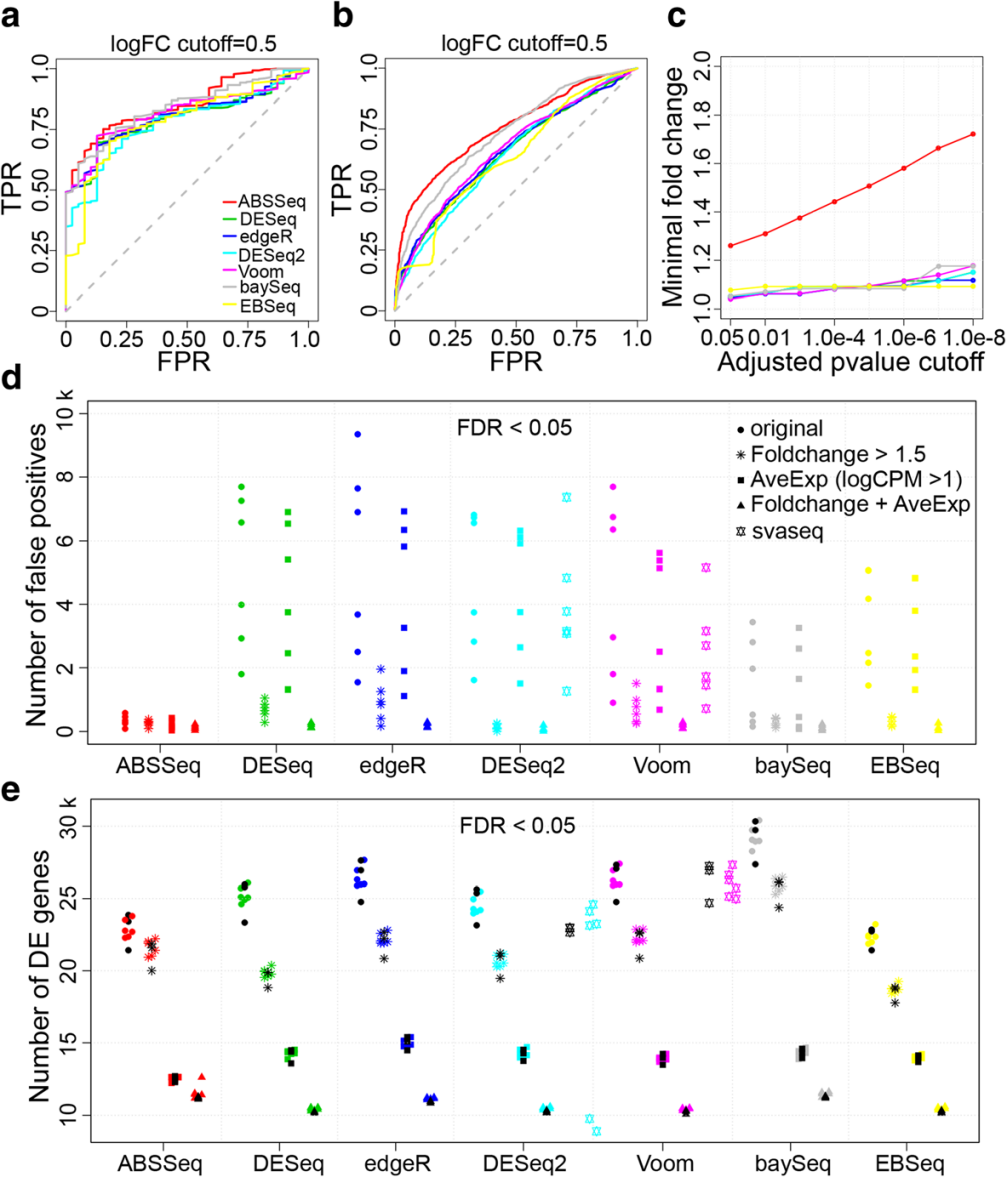
Comparison of methods using validated real data sets. **a**-**c** based on data from the MAQC study; **d**-**e** based on the ABRF data set. ROC analysis for (**a**) TaqMan and (**b**) PrimePCR data sets at a qRT-PCR absolute log-ratio (logFC) threshold of 0.5. TPR, true positive rate; FPR, false positive rate. ABSSeq performs better than other methods in detecting true differential expression. A gene was considered to be not differentially regulated if its logFC was less than 0.2. **c** Minimal fold changes under various ajusted *p*-value cutoffs for the MAQC II data set. **d** Number of false postives in comparisons of samples from same condition but different lab sites and (**e**) number of DE genes in comparison of samples from two conditons under additional filtering and confounding factor assessment approaches. Symbols in black show results from comparison of conditions from same laboratory and colored symbols those from comparison of conditions across laboratories. Genes are counted under 5 situations: orginal, without filtering (circle symbols); Foldchange, with a value greater than 1.5 (star symbols); AveExp, with average logCPM greater than 1 (square symbols); combination of Foldchange and AveExp (triangle symbols); and svaseq tested only for DESeq2 and Voom (pentacle symbols)

The results for both data sets are essentially identical (Figs. 3a and 3b). While the alternative methods can detect approximately half of the TaqMan validated DE genes without false positives, ABSSeq is even able to identify more than 75 % of the true DE genes with a false positive rate of less than 0.25 (Fig. 3a). ABSSeq reaches the highest AUC of 0.853 among six methods (baySeq: 0.840, Voom: 0.817, edgeR: 0.802, DESeq: 0.795, EBSeq: 0.783 and DESeq2: 0.777). For the PrimePCR data set, the AUC for each method decreases as the number of validated genes increases (Fig. 3b). Again, ABSSeq performs best among all seven methods, supporting its ability to discriminate efficiently between DE and non-DE genes.

Analysis approaches, which do not consider the magnitude of expression differences, might yield highly statistically significant DE for genes with only small fold change (as shown in Fig. 3c), which may however often be the result of chance. The number of these type of DE genes is usually not reduced by using an adjusted *p*-value cutoff in the alternative approaches, even if the cutoff is below 1.0e-8. Therefore, other cutoff criteria are required such as fold change, which has the problem that the biologically relevant cutoff point is not clear. The ABSSeq-based analysis instead produces high correlation between the minimal fold change and the inferred adjusted *p*-value, indicating that the *p*-value alone will select against DE genes with small fold change. Additional cutoff criteria therefore do not seem to be necessary for reliable DE gene identification.

### Influence of cut-off criteria and confounding factor analysis procedures

We next investigate the influence of additional cut-off criteria on DE detection with the help of the ABRF data set, which is based on RNA-Seq data generated for the same sample in three different laboratories. We apply the considered methods on this data set, which only contains variation caused by differences among the conisdered laboratories, such as biases during library preparation [28], but not true DE, thus allowing us to assess the efficacy of the methods to reduce the number of false positives ([28]; see also above). In spite of varying numbers of detected DE genes, ABSSeq reports lowest number of false positives among all methods, irrespective of any additional filtering approach (Fig. 3d). baySeq and EBSeq also produce small numbers of false positives then compared to the remaining methods excluding ABSSeq. For all methods, the number of false positives reduces dramatically when filtered by foldchange (>1.5; star symbols in Fig. 3c) but less so when filtered by expression level (AveExp, logCPM > 1; square symbols in Fig. 3d). This finding strongly suggests that a high foldchange cut-off increases power to control the false positive rate, yet with the problem that the choice of cut-off value will usually be arbitrary.

In addition, high specificity (i.e., efficient control of the false positive rate) might lead to low senstitivity (i.e., reduced efficiency to detect true positives). To evaluate the ability of ABSSeq to detect true positives, we apply ABSSeq and alternative methods on the ABRF data set whereby in this case we focus on the comparison of the two considered conditions (i.e., tissues) either within the considered laboratories (i.e., condition A and B from the same laboratory are compared) or across the laboratories (i.e., condition A from laboratory 1 is compared with condition B from laboratory 2, and so on for all possible combinations between the two conditions). The results are shown in Fig. 3e (black for comparison of conditions from same laboratory and other colors for comparison of conditions across laboratories). All seven methods report similar numbers of DE genes, especially after fold-change filtering. This result indicates that ABSSeq retains similar sensitivity than that shown by the alternative approaches.

Confounding variation can originate from library preparation or other kinds of batch effects. To remove its influence on DE detection, it can be modeled and thus integrated into the statistical analysis [28], as implemented in svaseq [37]. To illustrate the possible influence of such variation, we applied svaseq togethor with DESeq2 and Voom. Svaseq together with Voom is able to remove more than 50 % false positves for the ABRF data set (Fig. 3d, indicated by the pink pentacle symbols), in consistency with the previous application of the svaseq approach on data from the SEQC study [28]. However, when svaseq is combined with DESeq2 it leads to only a small decrease in the number of false positives (Fig. 3d, indicated by light blue pentacle symbols). This result may suggest that the performance of svaseq depends on the DE detection method itself and/or the linear model used in such methods. Moreover, the application of svaseq does not decrease sensitivity when combined with Voom and only to a small extent when combined with DESeq2 (Fig. 3e), suggesting that svaseq mainly improves removal of false positives but does not bias detection of true DE. In general, the usage of such confounding factor assessment procedures, including svaseq and also PEER [38] can help improve DE detection. Yet, at the moment, its combination with the various DE analysis methods is not straightforward, because both svaseq and PEER produce non-integer values, whereas several of the current DE analysis methods (including ABSSeq) rely on integer count data. It thus represents a promising challenge to further develop these procedures as integrated modules of the common DE detection methods.

### Assessment of statistical power via signal to noise ratio

**Fig. 4:**
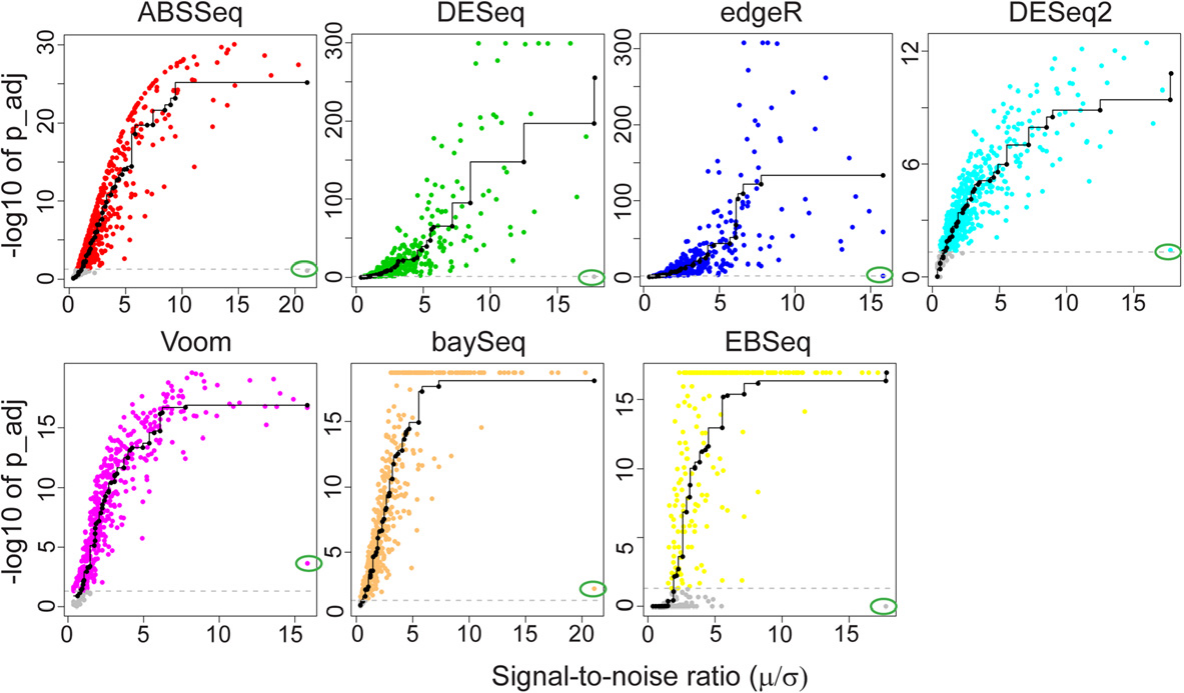
Correlation between signal-to-noise ratio and *p*-value with true DE present in only one condition. Evaluation is based on a total of 1514 genes that are exclusively expressed in one condition in the MAQC-II data set. Gray points indicate genes with adjusted *p*-value value ≥ 0.05. The data point highlighted by the green elipse refers to the gene with high signal-to-noise ratio but low expression. The correlation is inferred using isotonic regression (black line)

To evaluate the statistical power of each method in measuring the magnitude of DE in dependence of its variance, we repeated above comparison using genes that are exclusively expressed in only one condition of the MAQC-II data set following the approach from [20]. The magnitude of DE of genes expressed in only one condition is ideally shown as a signal-to-noise (SNR) ratio (mean over standard deviation), which should be monotonically correlated with the *p*-value [20]. A poor correlation between SNR ratio and *p*-value might lead to reduced sensitivity (type II error) by assigning a large *p*-value to small SNR ratio (i.e. high variance). The monotonic dependency between predictor (SNR ratio) and response (adjusted *p*-value) is inferred through an isotonic regression on 1514 paired variables (genes). Results are shown in Fig. 4. All methods but DESeq and edgeR exhibit the desired monotonic behavior between SNR ratio and adjusted p-value, in consistency with previous results from [20]. Two empirical Bayes based approaches: baySeq and EBSeq yield quite similar correlations between SNR ratio and posterior probabilities. In addition, Voom assigns a more significant adjusted *p*-value for one specific gene with high SNR ratio but low expression (marked by green elipse in Fig. 4) whereas alternative methods produce adjusted *p*-values of around 0.05 (gray dashed line), suggesting Voom is more sensitive to DE at low expression level. Since DESeq2 and Voom test DE on log fold change, we postulate that the closer correlation between SNR ratio and adjusted *p*-value of ABSSeq is due to modelling directly the magnitude of DE difference. Overall, these results suggest that ABSSeq seems to model the magnitude of count difference with higher accuracy, which might help DE inference by reducing false positives.

### Differential expression analysis of real data with unbalanced designs

Another real data set (HapMap-CEU) is taken from [39], consisting of 41 highly dispersed cDNA samples from 17 females and 24 males. DE genes are inferred from male–female comparisons. Following [23], a sensitivity analysis is predicted to find an over-representation of inferred DE genes from the sex chromosomes. Indeed, the top ten DE genes always include genes from sex chromosomes (Table 1). All methods except ABSSeq and Voom identify DE genes beyond sex chromosomes. This may indicate that ABSSeq and Voom retain higher specificity than the remaining methods and that alternative methods may not well model variance introduced by unequal smaple sizes. EBSeq produces the lowest number of DE genes from sex chromosomes but the highest number from autosomes, confirming the previously observed lack of power of this method for the analysis of data with such high dispersion and uneven sample sizes [13]. Given the unequal sample size in this data set, the similar performance of ABSSeq to that of alternative methods also suggests that our model is able to handle unequal sample sizes and high dispersion. In particular, in ABSSeq, we attempted to compensate for unequal sample size by adding expected reads counts to the smaller group until sample sizes are equal. We always take the mean reads count of the small group as expected count, in order to minimize possible biases in subsequent variance estimations (see also Methods). This compensation step is likely crucial for unbalanced data designs, especially in case of even larger differences than in our test data set. In the future, it may be worth exploring in more detail alternative compensation procedures.

**Table 1:** Number of DE genes from sex chromosomes detected by each method in the HapMap-CEU data set at FDR-ajusted *p*-value of 0.05

Method	Sex/Total	Sex in Top 10
ABSSeq	7/7	7
DESeq	7/25	5
edgeR	7/20	7
DESeq2	7/10	7
Voom	7/7	7
baySeq	9/27	7
EBSeq	2/38	2

### Moderating fold change

**Fig. 5:**
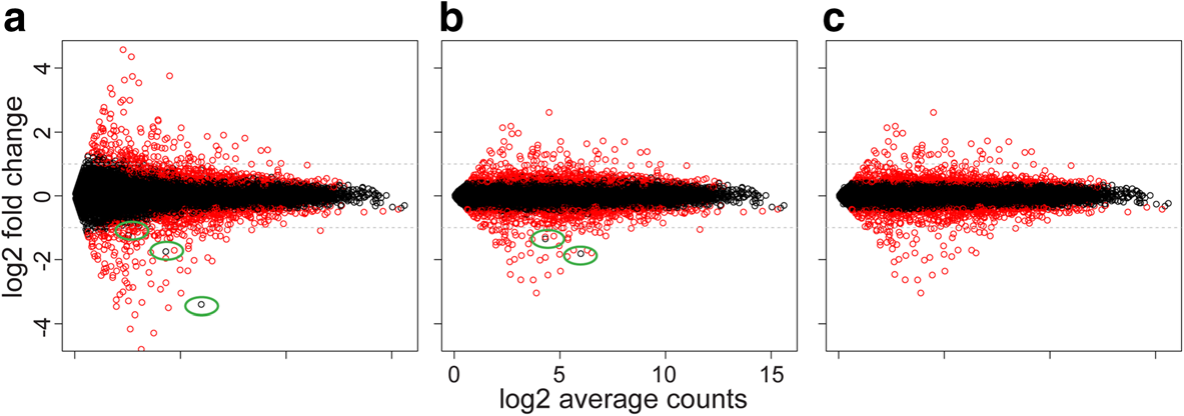
Moderation of log2 fold change. **a** Raw data (without shrinkage) of the Bottomfly study. **b** The same data corrected by expression level. **c** The same data corrected by expression level and gene-specific dispersion. DE genes (adjusted pvalue <0.05) are shown in red. Non-DE genes with high log2 fold change are marked by green elipses

Fold change often serves as a more informative indicator for biologists to identify DEs. It is also utilized in gene ranking to select candidates for further investigation and visualization (e,g, heatmap of several comparisons). However, the fold change neglects variance across samples and might not necessarily be informative, especially for genes with low counts (see also discussion above). To overcome this problem, DESeq2 introduces an empirical Bayes shrinkage for fold change estimation, which moderates the log fold change according to gene-specific dispersion [12]. Fold change can also be represented as a function of absolute count differences (see Methods), suggesting a potential moderation of fold change via counts difference (e.g., expected counts difference). Therefore, we introduce a fold change shrinkage procedure according to count differences and dispersion. Figure 5 shows how it works using the Bottomly data set [40]. Genes with small counts tend to have high raw fold changes (Fig. 5a), which constrains reliable gene ranking by fold change at dynamic expression level. Shrinking fold change by adding pseudocounts according to expression level (see Methods) removes this trend (Fig. 5b).

However, this shrinkage approach neglects the gene-specific dispersion and thus shows no effects on non-DE genes with high dispersion as well as high expression (Fig. 5a and b, marked by green elipses). After taking account of gene-specific dispersion (Fig. 5c), the fold changes approximately reflect DE genes (in red under adjusted *p*value <0.05) and produce nearly evenly distributed fold change values, apparently improving gene ranking and visualization. Notably, shrinkage with gene-specific dispersion only influences a small part of genes with high dispersion. Unlike the approach in DESeq2, our shrinkage method on gene-specific dispersion is based on *p*-values and therefore does not change the number of inferred significant DE genes. In practice, users can obtain all three types of fold change values (raw, shrinked by smoothed dispersion according to mean, and shrinked by both smoothed and gene-specific dispersions) in ABSSeq.

## Conclusions

Here we introduce a new method for differiential expression analysis of RNA-Seq count data, ABSSeq. Distinct from other current methods, ABSSeq infers DE genes through the absolute differences in gene expression and assumes the differences to be influenced by two sources of variation: that found for average gene expression levels and that found for the magnitude of differential expression. Our approach employs a NB distribution to model these two parts and, as a consequence, it is able to detect DE genes more effectively than existing methods, as demonstrated by our analysis of both real and simulated data. In particular, ABSSeq shows an advantage in discriminating DE genes against non-DE ones, it applies an efficient outlier detection approach and is thus robust against outliers. Morever, ABSSeq inferred *p*-values correlate with the magnitude of count differences, thus producing a linear relationship between SNR ratio and *p*-value. As a result, it reduces type I error rates at both very low and high expression level and it also leads to a smaller number of highly significant DE genes with small fold change.

In addition, ABSSeq introduces a procedure to shrink fold change according to the smoothed dispersion across expression level and observed dispersion (gene-specific), which permits fold change comparisons across genes and thus might favor downstream analyses, such as gene set enrichment analysis by ranking [41], clustering and visualization (heatmap) or candidate selection. A potential improvement of our approach in the future may be to adapt it to allow usage of more complex models which consider multiple conditions, its combination with additional normalization procedures, such as those implemented in PEER and svaseq [37, 38], which can further help to filter out unwanted variation, and also adjustment of our approach to allow for analysis of DE at the transcript level (in addition to the gene level, currently implemented). In summary, based on our analysis, we conclude that ABSSeq represents a highly efficient approach for identification of significant DEs across a wide range of conditions and may help efficient downstram analysis of DEs.

## Methods

### Datasets

In this study, the performance of methods is assessed with the help of two types of data sets: simulated and real. The simulated data sets are derived from the study of Soneson et al. [7]. Following the approach in [21], Soneson et al. used the mean and variances from Pickrell’s RNA-Seq dataset ([42]; 69 lymphoblastoid human cell lines derived from unrelated Nigerian individuals) as parameters to generate read counts for each gene from a Poisson or NB distribution. The simulated data sets were generated to follow either a NB distribution (denoted by NB), half NB and half Poisson (denoted by P), NB with single sample outliers (denoted by S) and NB with random outliers (denoted by R). Each set includes 10 independently repeated simulations of two treatment groups and different replicate sample sizes of 2, 5 or 10 for each group. A total of 12,500 genes with high expression (reads count) is considered, for which expression variation is simulated with or without DE genes according to the tests performed.

Five real data sets were considered. Four of these (all except of ABRF) were downloaded from http://bowtie-bio.sourceforge.net/recount/ [43]. The MicroArray Quality Control (MAQC) study has been used to evaluate the performance of different gene expression analysis methods [36]. It is based on replicated RNA samples of the human whole body (UHR) and brain (BHR) [44, 45]. We use the MAQC II data set for analysis of performance of DE detection methods. For each group (body or brain), seven technical replicates are produced. We filtered out genes with zero read counts across samples before analysis. The raw data of MAQC-II are available from the NCBI SRA database under SRA010153. Moreover, we also use two qRT-PCR validated data sets, either based on the TaqMan methodology, comprising more than 1000 genes from MAQC I, available at NCBI Gene expression Omnibus database under GSE5350, and that based on PrimePCR including more than 20,000 genes from SEQC (MAQC III), available under GSE56457.

The modencodefly data set served to study gene expression during the development of *Drosophila melanogaster* [26], covering 30 distinct developmental stages. Each of the stages consists of 4 up to 6 technical replicates. We subsample from each stage 4 replicates to construct a 2:2 pairwise study.

The HapMap-CEU data set [39] includes 41 samples based on immortalized B-cells from 41 unrelated CEPH grandparents. It contains 17 female samples and 24 male samples.

The ABRF data set is the Association of Biomolecular Resource Facilities next-generation sequencing (ABRF-NGS) study on RNA-seq, which aims to assess RNA-Seq data across laboratory sites and platforms [27] and relies on the the same samples used in the Sequencing Quality Control (SEQC) study [29]. Here we use data from two samples generated via a ribo-depleted protocol, namely RNA from cancer cell lines and also RNA from pooled normal human brain tissues. We thus exclude data from mixtures of these samples and that based on other protocols. The raw data and counts tables are available at the Gene Expression Omnibus database under accession number GSE48035. This study compared RNA-Seq data for the same samples assessed in different laboratories.

The Bottomly data set is from a study that characterized transcriptomic differences between two inbred mouse strains (C57BL/6J and DBA/2J) with 10 and 11 replicates each, respectively [40]. We filtered out genes with zero read counts across samples before analysis.

### Data structure and normalization

RNA-Seq data is represented as count of reads (*c*_*ij*_) for genes (*i*) and samples (*j*) at different conditions (A, B or more), which are discrete. Due to technical and other reasons, the total number of reads varies between samples or even sequencing lanes. The read count must thus be normalized before comparison across samples. The most common practice is to scale the counts according to the total number of reads of each sample [46, 47]. However, this approach was shown to introduce a bias in DE inference since DE genes can be responsive for large variations in total read number [36]. Here, for ABSSeq, we chose the quantile-based procedure, which yielded much better concordance with the qRT-PCR data [36]. In addition, we also offer geometric mean based normalization procedure in ABSSeq, which we borrowed from DESeq.

### Outlier detection and replacement

Outliers mask the statistical significance by influencing the estimation of mean and variance. Given extreme high read counts outliers are often present in one or more RNA-Seq samples and thus it is essential for DE inference to reduce the impact of outliers [12, 13, 48]. Since RNA-Seq data could be treated to be log-normally distributed, ABSSeq utilizes the median absolute deviation (MAD) to detect the outliers in log-transformed read counts. However, due to typically limited sample size in RNA-Seq data, MAD could be extremely small or even zero possibly resulting in over-detection. To solve this problem, we adjusted the MAD of each gene using the highest population standard deviation (SD) *σ*_0_, that is

1 $$ {\widehat{M}}_{iA\left|\right|B}=\sqrt{\frac{n_{A\left|\right|B}{M}_{iA\left|\right|B}^2+{n}_0{\sigma}_0^2}{n_{A\left|\right|B}+{n}_0}} $$

where *n*_*A*||*B*_ is the sample size for each condition and *n*_0_ is the weight for *σ*_0_. It is similar to empirical Bayes in limma and shrinks the observed MAD toward the highest population SD, thus avoiding small MADs in further analyses. In practice, we set *n*_0_ = 2 and *σ*_0_ = *σ*_*μ*=1_ due to the quadratic mean-variance relationship in RNA-Seq data (highest dispersion at lowest expression level), and also provide an interface for the user to change these two values. Thus, the outliers are defined as

2 $$ \log \left({c}_{i,j\in A\left|\right|B}+1\right)- median\left( log\left({c}_{i,j\in A\left|\right|B}+1\right)\right)-2{\widehat{M}}_{iA\left|\right|B}>0 $$

and replaced by $median\left( log\left({c}_{i,j\in A\left|\right|B}+1\right)\right)+{\widehat{M}}_{iA\left|\right|B}$. The natural exponent of the read counts after outlier replacement is then used as input for DE testing in ABSSeq.

### Inferring DE genes based on absolute expression differences between conditions

DE inference relies on an assessment of the difference of expression levels between two conditions (or more) as well as the variance across replicate samples. The popular null hypothesis for testing DE is that the mean read count for a particular gene is identical between conditions. However, the standard analysis of such a hypothesis neglects the magnitude of encountered differences. Here we use a distinct test statistic: the absolute difference of read counts between conditions (specifically, A and B), which was firstly applied to detect differential expression, epigenetics changes and transcription factors binding sites in the program EpiCenter [10], that is

3 $$ {D}_i=\left|{\displaystyle \sum_{j\in A}{c}_{ij}}-{\displaystyle \sum_{j\in B}{c}_{ij}}\right| $$

When the sample sizes between groups are not equal, *D*_*i*_ introduces a bias by favoring the larger group, which has a higher likelihood to reach higher sum counts by chance, thus more likely resulting in non-zero *D*_*i*_). For this reason, ABSSeq compensates the smaller group with the most likely read counts: the mean. In these cases, *D*_*i*_ might not be an integer and needs to be rounded to the nearest integer.

*D*_*i*_ is always discrete and apparently overdispersed as *D*_*i*_ inherits variance from ${\displaystyle \sum_j{c}_{ij}}$ and is less than ${\displaystyle \sum_j{c}_{ij}}$ (${\displaystyle \sum_j{c}_{ij}}$ is overdispersed [1, 3]), which suggests that it follows a NB distribution. Based on this idea, ABSSeq employs a NB distribution to model *D*_*i*_, which has two parameters, the mean *m*_*i*_ and size factor *r*_*i*_, that is

4 $$ {D}_i\sim \operatorname{NB}\left({m}_i,{r}_i\right) $$

*m*_*i*_ can be treated as the expected value or baseline of *D*_*i*_ which is proportional to average expression level (larger expected value of *D*_*i*_ at higher expression level) or determined using the coefficient of variation (CV) in the tested data. Therefore, *m*_*i*_ is

5 $$ {m}_i=\alpha {c}_i $$

where *c*_*i*_ and *α* are larger value of sum counts, general CV. *r*_*i*_ as the size factor is dependent on the mean-variance relationship and determines the scale of information contained by*D*_*i*_. We assume*D*_*i*_ to inherit dispersion from *c*_*i*_ (i.e., the shape of its distribution is similar to that of *c*_*i*_). As *c*_*i*_ could be written as *c*_*i*_ = *nμ*_*i*_ *n* = max(*n*_*A*_, *n*_*B*_), *μ*_*i*_ = max(*μ*_*iA*_, *μ*_*iB*_) (the *μ*_*A*||*B*_ denotes mean of each condition), we assume *c*_*i*_ has the same dispersion under *μ*_*i*_. Therefore, the dispersion of *c*_*i*_ becomes

6 $$ {v}_i=\frac{\left({s}_{iA}^2+{s}_{iB}^2\right)-{\mu}_i}{\mu_i^2} $$

whereby *v*_*i*_ and *s*_*iA*||*B*_^2^ denote the pooled dispersion factor, the mean and variance of each condition, respectively. *r*_*i*_ is then given as

7 $$ {r}_i=1/{v}_i $$

As a result, DE detection is based on the magnitude of *D*_*i*_ against its expected value *m*_*i*_ and dispersion *r*_*i*_. ABSSeq also allows DE detection on paired samples by replacing *s*_*iA*_^2^ + *s*_*iB*_^2^ with variance drived directly from paired differences.

### Moderating *m*_*i*_, *r*_*i*_

It is well-known that the mean-variance relationship of RNA-Seq data is basically quadratic [5], which suggests a relative higher uncertainty of *c*_*i*_ (higher *m*_*i*_) for genes with low expression levels. To account for the dynamic uncertainty, we moderated *m*_*i*_ by adding pseudocounts to *c*_*ij*_ according to the mean-variance relationship, which has no influence on*D*_*i*_ and *s*_*iA*||*B*_^2^ but *μ*_*i*_ and *r*_*i*_, that is

8 $$ {\widehat{\mu}}_i={\mu}_i+{\mu}_{0i}\ {\widehat{c}}_i={c}_i+n{\mu}_{0i} $$

${\widehat{\mu}}_i$ indeedly represents the upper bound of *μ*_*i*_. To estimate *μ*_0*i*_, we firstly construct the mean-variance relationship by applying local regression [49] on the graph $\left(\sqrt{v_i},\ {\mu}_i\right)$ with *locfit* package from R, which has been introduced by DESeq. That is

9 $$ {\widehat{v}}_i=f{\left(\sqrt{v_i}\right)}^2 $$

Then the smoothed or expected variance for each gene is given by

10 $$ {\widehat{s}}_i^2={\mu}_i+{\widehat{v}}_i{\mu}_i^2 $$

Since the uncertainty of *μ*_*i*_ always decreases as the expression level or sample size increases, we assume that ${\widehat{\mu}}_{0i}$ could be written as

11 $$ {\mu}_{0i}=\sqrt{\frac{\theta }{\mu_i}\frac{{\widehat{s}}_i^2}{n-1}}\kern0.75em \theta =\sqrt{mean\left(\frac{s_{iA}^2+{s}_{iB}^2}{2}\right)} $$

where *θ* serves as background of uncertainty across all genes.

When the observed variance is 0 (i.e., *c*_*ij*_ is the same in all samples), the dispersion of sum counts *c*_*i*_ simply becomes ${\widehat{v}}_i/n$ (combined NB distributed variables with sum size factor $n/{\widehat{v}}_i$), which suggests ${\widehat{v}}_i/n$ serves as the background of *v*_*i*_. However, ${\widehat{v}}_i$ is usually obained from part of the tested data (on *v*_*i*_ > 0), indicating underestimation of ${\widehat{v}}_i$. To penalize this, we add a basic dispersion factor *v*_0_ to *v*_*i*_, which becomes

12 $$ {\widehat{r}}_i=\frac{1}{{\overline{v}}_i}\kern0.75em {\overline{v}}_i={v}_0+{v}_i+{\widehat{v}}_i/n $$

*v*_0_ is determined by quantile estimation on *v*_*i*_ with *v*_*i*_ > 0, that is

13 $$ {v}_0= quantile\left({v}_i\Big|{v}_i>0,\sqrt{\beta}\right) $$

where *β* is the percentage of *v*_*i*_ on *v*_*i*_ < 0. Generally, it permits a smaller *v*_0_ for lower *β*.

Notably, the small variance of *μ*_*i*_ (*s*_*iA*_^2^ + *s*_*iB*_^2^ ≤ *μ*_*i*_) is not caused by *r*_*i*_. However, neglecting this variance will introduce false positives at low expression level since the small variance has a higher impact on *μ*_*i*_ when *μ*_*i*_ is small. On the other hand, this small variance could be treated as noise for *μ*_*i*_ or *c*_*i*_. In light of this, we add a small value to *c*_*i*_. *m*_*i*_ becomes

14 $$ {\widehat{m}}_i=\alpha \left({\widehat{c}}_i+\varepsilon \right)\kern0.5em \varepsilon =\sqrt{n_i \max \left({s}_{iA}^2+{s}_{iB}^2,{\mu}_i\right)} $$

### Estimating *α*

After shifting read counts according to the mean-variance relationship, we simply assume that CVs of all genes are identical. While the SD of log-transformed data stands for the CV at original scale, we get *α* by

15 $$ \alpha = mean\left({\sigma}_i\right) $$

where *σ*_*i*_ is obtained by fitting a linear model to log-transformed counts from limma. In practice, the estimated *α* usually ranges from 0.1 to 0,3. *α* could also be provided by the user (i.e., testing DEs on prior threshold).

#### *P*-value calling

Following (2), we can calculate the *p*-value for each gene by the cumulative distribution function of $\operatorname{NB}\left({\widehat{m}}_i,{\widehat{r}}_i\right)$. The false discovery rate (FDR) by Benjamini-Hochberg is used to account for multiple testing as a default.

### Moderating log fold change

The log fold change can be described as

16 $$ F{C}_i= \log \left(\frac{c_i}{c_i-{D}_i}\right) $$

Thus, we can moderate it busing *c*_*i*_ or *D*_*i*_. Indeed, in (7), we moderate *c*_*i*_ by adding pseudocounts, which mainly shrinks fold change in response to uncertainity across expression level but not gene-specific dispersion (observed). The gene-specific dispersion ${\widehat{r}}_i$ also determines the scale of information contained by fold change, i.e. a high dispersion indicates low information of fold change, and vice versa [12]. On the other hand, ${\widehat{r}}_i$ also controls the information contained by *D*_*i*_, indicating a possible moderation of *D*_*i*_ as well as fold change by shrinkage of ${\widehat{r}}_i$. Under certain pvalue from $\operatorname{NB}\left({\widehat{m}}_i,{\widehat{r}}_i\right)$, increasing ${\widehat{r}}_i$ (decreasing dispersion) will reduce expectation of *D*_*i*_ and thus fold change. Based on this idea, we obain a new *D*_*i*_ by replacing ${\widehat{r}}_i$ with the dispersion obtained through the probability quantile function from the NB distribution, that is

17 $$ {\widehat{D}}_i=q\operatorname{NB}\left({p}_i,{\widehat{m}}_i,{r}_0\right)\kern0.5em {r}_0= \max \left({\overline{r}}_i,1/ mean\left({\overline{v}}_i\right)\right) $$

where *p*_*i*_ is the *p*value for gene *i*. Notably, the moderation is only applied on genes with ${\widehat{r}}_i$ less than *r*_0_. Using this approach the log fold change is then calculated by (16) with *ĉ*_*i*_ and ${\widehat{D}}_i$, which approximately normalizes fold change toward the common dispersion (mean). In addition, we also provide an interface for user to change *r*_0_.

## Software tools

The figures in this study have been plotted using R.

## Abbreviations

AUC, area under curve; DE, differential expression; FC, fold change; FDR, false discovery rate; FPR, false positve rate; logFC, log2 of fold change; NB, negative binomial; RNA-Seq, (high-throughput) sequencing of RNA; ROC, receiver operating characteristic; SEQC, sequencing quality control; TPR, true positive rate

## Additional files

Additional file 1: Overview and command lines for differential expression analysis in R. (PDF 15 kb) Additional file 2: Tables S1 and S2. On the results of the statistical comparison of differential expression analysis methods. (PDF 322 kb) Additional file 3: Method-dependent variation in type I error on ABRF data set. (PDF 314 kb)
