# Supplementary material for: ABSSeq: a new RNA-Seq analysis method based on modelling absolute expression differences
Source: BMC Genomics. 2016 Aug 4;17:541. doi: 10.1186/s12864-016-2848-2 (PMC4973090; doi:10.1186/s12864-016-2848-2)
Supplement: Additional file 1: — Overview and command lines for differential expression analysis in R. (PDF 15 kb) [file 12864_2016_2848_MOESM1_ESM.pdf]

R commands

Here, we list the R commands for each method that were used to analyze the data in this study. As explained in the main text, we run each method in default settings. All analyses were performed with R version 3.2.2. The data matrix and conditions are denoted as cdat and cgroup, respectively.

ABSSeq

The ABSSeq package, version 1.6.1, is available in Bioconductor.

```
>  library(ABSSeq)
>  obj  <- ABSDataSet( cdat, cgroup )
>  obj <- ABSSeq(obj)
> res <- results(obj, c("Amean","Bmean","foldChange","pvalue","adj.pvalue"))
```

DESeq

The DESeq package, version 1.20.0, can be obtained from Bioconductor.

```
>  library(DESeq)
>  cds <- newCountDataSet(cdat, cgroup)
>  cds <- estimateSizeFactors(cds)
>  if(length(cgroup) == 2){
+  cds <- estimateDispersions(cds, method= "blind", fitType='local', sharingMode='fit-only')
+    }else{
+      cds <- estimateDispersions(cds, method= "per-condition", fitType='local')
+    }
>  res <- nbinomTest(cds, "condA", "condB")
```

edgeR

The edgeR package, version 3.10.2, can be obtained from Bioconductor.

```
>  library(edgeR)
>  y <- DGEList(counts=cdat, group=cgroup)
>  y <- calcNormFactors(y)
>  if(2 == length(cgroup)){
+        y$common.dispersion = 0.4
+    }else{
+      y <- estimateCommonDisp(y)
+      y <- estimateTagwiseDisp(y)
+    }
>  et <- exactTest(y)
>  res <- topTags(et,n=nrow(et))
```

DESeq2

The DESeq2 package, version 1.8.1, can be obained from Bioconductor.

```
> library(DESeq2)
> ds <- DESeqDataSetFromMatrix(countData = cdat, colData = data.frame(cgroup), design =
+                                               ~ cgroup)
> ds <- DESeq(ds)
> res <- results(ds)
```

limma

The limma package, version 3.24.15, can be obtained from Bioconductor. It has been evaluated with two different settings: Voom and QN.

Here is Voom.

```
> library(limma)
> library(edgeR)
> design <- model.matrix(~0+cgroup)
> colnames(design) <- levels(cgroup)
> nf <- calcNormFactors(cdat)
> dat <- voom(cdat, design, plot=FALSE, lib.size=colSums(cdat) * nf)
> fit <- lmFit(dat,design)
> contrast.matrix <- makeContrasts("condB - condA", levels=design)
> fit2 <- contrasts.fit(fit, contrast.matrix)
> fit2 <- eBayes(fit2)
> res=decideTests(fit2,p.value=q.cut,lfc=lfc)
> tab<-topTable(fit2, adjust = "BH", number=nrow(fit2), sort.by='logFC')
```

Here is QN.

```
> library(limma)
> design <- model.matrix(~0+cgroup)
> colnames(design) <- levels(cgroup)
> dat <- log2(cdat+1)
> dat <- normalizeBetweenArrays(dat,method='quantile')
> fit <- lmFit(dat,design)
> contrast.matrix <- makeContrasts("condB - condA", levels=design)
> fit2 <- contrasts.fit(fit, contrast.matrix)
> fit2 <- eBayes(fit2)
> res=decideTests(fit2,p.value=q.cut,lfc=lfc)
> tab<-topTable(fit2, adjust = "BH", number=nrow(fit2), sort.by='logFC')
```

baySeq

The baySeq package, version 2.2.0, can be obtained from Bioconductor.

```
> library(baySeq)
> cl=NULL
```

```
>  bcd <- new("countData", data = cdat, replicates = cgroup,
+                    groups = list(NDE = rep(1, length(cgroup)), DE = cgroup))
>  bcd@libsizes <- getLibsizes(bcd)
>  bcd <- getPriors.NB(bcd,smaplesize=5000, cl = cl)
>  bcd <- getLikelihoods.NB(bcd, cl = cl)
>  baySeq.posteriors.DE <- exp(bcd@posteriors)[, 2]
>  baySeq.FDR <- topCounts(bcd, group = 'DE', FDR = 1)$FDR[match(rownames(cdat),
+                                              rownames(topCounts(bcd, group = 'DE', FDR = 1)))]
```

EBSeq

The EBSeq package, version 1.10.0, can be obtained from Bioconductor.

```
>  library(EBSeq)
>  Sizes <- MedianNorm(cdat)
>  bcd <- EBTest(Data=cdat, Conditions=as.factor(cgroup),
+          sizeFactors=Sizes,maxround=5)
>  res <- GetDEResults(bcd)
```
