# Supplementary material for: ABSSeq: a new RNA-Seq analysis method based on modelling absolute expression differences
Source: BMC Genomics. 2016 Aug 4;17:541. doi: 10.1186/s12864-016-2848-2 (PMC4973090; doi:10.1186/s12864-016-2848-2)
Supplement: Additional file 2: Tables S1 and S2. — On the results of the statistical comparison of differential expression analysis methods. (PDF 322 kb) [file 12864_2016_2848_MOESM2_ESM.pdf]

## Table S1. Multiple comparisons of type I error performance of all methods

| | Tukey multiple comparisons of Means | | | | | | Wilcoxon tests |
|---|---|---|---|---|---|---|---|
| | NB | | | R | | | Modencodefly data set |
| | N=2 | N=5 | N=10 | N=2 | N=5 | N=10 | |
| **Paired comparison** | **p adj** | **p adj** | **p adj** | **p adj** | **p adj** | **p adj** | **p adj** |
| DESeq-ABSSeq | 0.00E+00 | 0.00E+00 | 0.00E+00 | 0.00E+00 | 0.00E+00 | 0.00E+00 | 2.21E-10 |
| DESeq2-ABSSeq | 0.00E+00 | 0.00E+00 | 0.00E+00 | 0.00E+00 | 0.00E+00 | 0.00E+00 | 3.34E-03 |
| edgeR-ABSSeq | 0.00E+00 | 0.00E+00 | 0.00E+00 | 0.00E+00 | 0.00E+00 | 0.00E+00 | 2.21E-10 |
| Voom-ABSSeq | 0.00E+00 | 0.00E+00 | 0.00E+00 | 0.00E+00 | 0.00E+00 | 0.00E+00 | 2.21E-10 |
| DESeq2-DESeq | 0.00E+00 | 0.00E+00 | 0.00E+00 | 5.11E-05 | 9.81E-01 | 0.00E+00 | 2.08E-05 |
| edgeR-DESeq | 0.00E+00 | 0.00E+00 | 0.00E+00 | 8.51E-01 | 0.00E+00 | 0.00E+00 | 9.19E-02 |
| Voom-DESeq | 1.70E-06 | 0.00E+00 | 0.00E+00 | 0.00E+00 | 0.00E+00 | 0.00E+00 | 3.32E-03 |
| edgeR-DESeq2 | 1.00E+00 | 0.00E+00 | 0.00E+00 | 0.00E+00 | 0.00E+00 | 0.00E+00 | 5.56E-07 |
| Voom-DESeq2 | 3.80E-06 | 0.00E+00 | 6.93E-02 | 0.00E+00 | 0.00E+00 | 0.00E+00 | 2.21E-08 |
| Voom-edgeR | 4.20E-06 | 1.81E-04 | 0.00E+00 | 0.00E+00 | 0.00E+00 | 0.00E+00 | 9.19E-02 |
| edgeR-robust-edgeR | / | / | / | 0.00E+00 | 0.00E+00 | 1.97E-02 | / |
| edgeR-robust-ABSSeq | / | / | / | 0.00E+00 | 0.00E+00 | 0.00E+00 | / |
| edgeR-robust-Voom | / | / | / | 0.00E+00 | 0.00E+00 | 0.00E+00 | / |
| edgeR-robust-DESeq | / | / | / | 0.00E+00 | 0.00E+00 | 0.00E+00 | / |
| edgeR-robust-DESeq2 | / | / | / | 0.00E+00 | 0.00E+00 | 0.00E+00 | / |

* edgeR-robust was only applied on data sets that contain outliers (indicated by R)

## Table S2. Tukey's multiple comparisons of means for AUCs

| | Tukey multiple comparisons of Means | | | | | |
|---|---|---|---|---|---|---|
| | NB | | | R | | |
| | N=2 | N=5 | N=10 | N=2 | N=5 | N=10 |
| **Paired comparison** | **p adj** | **p adj** | **p adj** | **p adj** | **p adj** | **p adj** |
| EBSeq-ABBSeq | 0.00E+00 | 0.00E+00 | 2.62E-02 | 0.00E+00 | 0.00E+00 | 0.00E+00 |
| baySeq-ABSSeq | 8.73E-01 | 9.08E-01 | 5.58E-02 | 9.99E-01 | 0.00E+00 | 0.00E+00 |
| DESeq-ABSSeq | 1.00E+00 | 9.98E-01 | 7.86E-01 | 1.30E-04 | 0.00E+00 | 0.00E+00 |
| DESeq2-ABSSeq | 0.00E+00 | 0.00E+00 | 0.00E+00 | 1.00E-03 | 0.00E+00 | 0.00E+00 |
| edgeR-ABSSeq | 1.00E+00 | 9.98E-01 | 1.00E+00 | 2.65E-02 | 0.00E+00 | 0.00E+00 |
| edgeR-robust-ABSSeq | / | / | / | 1.00E+00 | 0.00E+00 | 0.00E+00 |
| Voom-ABSSeq | 9.96E-01 | 3.05E-02 | 0.00E+00 | 9.68E-01 | 0.00E+00 | 0.00E+00 |
| EBSeq-baySeq | 0.00E+00 | 6.11E-03 | 1.00E+00 | 0.00E+00 | 4.71E-01 | 0.00E+00 |
| DESeq-baySeq | 9.15E-01 | 9.97E-01 | 6.97E-01 | 7.05E-04 | 0.00E+00 | 9.81E-01 |
| DESeq2-baySeq | 3.90E-06 | 0.00E+00 | 4.10E-06 | 4.77E-03 | 0.00E+00 | 0.00E+00 |
| edgeR-baySeq | 7.76E-01 | 6.09E-01 | 1.08E-01 | 8.94E-02 | 9.50E-02 | 8.78E-04 |
| edgeR-robust-baySeq | / | / | / | 9.99E-01 | 1.00E+00 | 9.70E-04 |
| Voom-baySeq | 9.95E-01 | 3.79E-01 | 3.08E-03 | 9.99E-01 | 0.00E+00 | 0.00E+00 |
| EBSeq-DESeq | 0.00E+00 | 8.58E-04 | 5.13E-01 | 2.25E-01 | 0.00E+00 | 0.00E+00 |
| DESeq2-DESeq | 0.00E+00 | 0.00E+00 | 0.00E+00 | 9.99E-01 | 9.12E-01 | 0.00E+00 |
| edgeR-DESeq | 1.00E+00 | 9.21E-01 | 9.09E-01 | 7.58E-01 | 1.00E-07 | 2.60E-05 |
| edgeR-robust-DESeq | / | / | / | 1.05E-04 | 0.00E+00 | 2.90E-05 |
| Voom-DESeq | 9.99E-01 | 1.21E-01 | 1.12E-05 | 4.93E-03 | 0.00E+00 | 0.00E+00 |
| EBSeq-DESeq2 | 1.40E-06 | 1.00E-07 | 1.22E-05 | 6.34E-02 | 0.00E+00 | 0.00E+00 |
| edgeR-DESeq2 | 0.00E+00 | 0.00E+00 | 0.00E+00 | 9.69E-01 | 0.00E+00 | 0.00E+00 |
| edgeR-robust-DESeq2 | / | / | / | 8.19E-04 | 0.00E+00 | 0.00E+00 |
| Voom-DESeq2 | 3.00E-07 | 0.00E+00 | 5.28E-01 | 2.72E-02 | 0.00E+00 | 0.00E+00 |
| EBSeq-edgeR | 0.00E+00 | 1.50E-05 | 5.42E-02 | 3.02E-03 | 1.53E-04 | 9.49E-02 |
| edgeR-robust-edgeR | / | / | / | 2.25E-02 | 3.01E-02 | 1.00E+00 |
| EBSeq-edgeR-robust | / | / | / | 0.00E+00 | 7.57E-01 | 8.86E-02 |
| EBSeq-Voom | 0.00E+00 | 6.15E-01 | 7.57E-03 | 4.00E-07 | 0.00E+00 | 0.00E+00 |
| Voom-edgeR | 9.84E-01 | 5.94E-03 | 1.00E-07 | 2.97E-01 | 0.00E+00 | 0.00E+00 |
| Voom-edgeR-robust | / | / | / | 9.57E-01 | 0.00E+00 | 0.00E+00 |

* edgeR-robust was only applied on data sets that contain outliers (indicated by R)
