# Supplementary material for: ABSSeq: a new RNA-Seq analysis method based on modelling absolute expression differences
Source: BMC Genomics. 2016 Aug 4;17:541. doi: 10.1186/s12864-016-2848-2 (PMC4973090; doi:10.1186/s12864-016-2848-2)
Supplement: Additional file 3: — Method-dependent variation in type I error on ABRF data set. (PDF 314 kb) [file 12864_2016_2848_MOESM3_ESM.pdf]

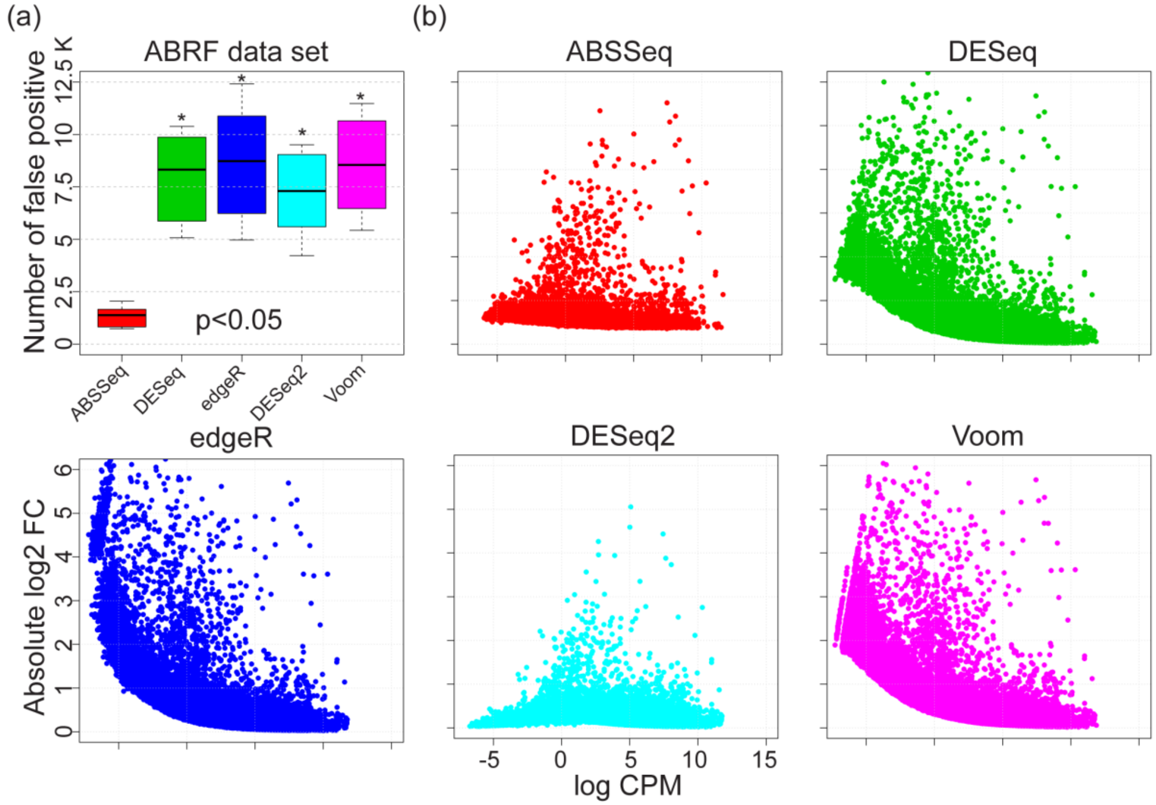

Figure S1. Method-dependent variation in type I error using the ABRF data set. (a) Number of DE genes reported by each method; (b) Points show the absolute log fold change (FC, y-axis) distribution of false positives against the expression level (logCPM, x-axis). DE calling uses data for the same sample generated by different laboratories. Only ABSSeq reports less than 5% DE genes (35766 in total) under $p < 0.05$. DESeq2 also reduces type I error at low expression level but not low fold-change.
